# Protective Effect of the Plant Extracts of *Erythroxylum* sp. against Toxic Effects Induced by the Venom of *Lachesis muta* Snake

**DOI:** 10.3390/molecules21101350

**Published:** 2016-10-11

**Authors:** Eduardo Coriolano de Oliveira, Rodrigo Alves Soares Cruz, Nayanna de Mello Amorim, Marcelo Guerra Santos, Luiz Carlos Simas Pereira Junior, Eladio Oswaldo Flores Sanchez, Caio Pinho Fernandes, Rafael Garrett, Leandro Machado Rocha, André Lopes Fuly

**Affiliations:** 1 Department of Molecular and Cellular Biology, Institute of Biology, Federal Fluminense University, Niterói 24020-141, RJ, Brazil; eduardocoriolano@globo.com (E.C.d.O.); nayanna_bio@hotmail.com (N.d.M.A.); jrbuzios@yahoo.com.br (L.C.S.P.J.); 2 Department of Biological and Health Sciences, Faculty of Pharmacy, Federal University of Amapá, Macapá 68903-419, AP, Brazil; rodrigo@unifap.br; 3 Faculdade de Formação de Professores, University of the State of Rio de Janeiro, Rio de Janeiro 24435-005, RJ, Brazil; marceloguerrasantos@gmail.com; 4 Laboratory of Biochemistry of Proteins from Animal Venoms, Research and Development Center, Ezequiel Dias Foundation, Belo Horizonte 30510-010, MG, Brazil; eladiooswaldo@gmail.com; 5 Laboratory of Phytopharmaceutical Nanobiotechnology, Department of Biological and Health Sciences, Federal University of Amapá, Macapá 68903-419, AP, Brazil; caio_pfernandes@yahoo.com.br; 6 Mass Spectrometry Laboratory, Institute of Chemistry, Federal University of Rio de Janeiro, Rio de Janeiro 21941-598, RJ, Brazil; rafael_garrett@iq.ufrj.br; 7 Department of Pharmaceutical Technology, Faculty of Pharmacy, Fluminense Federal University, Niterói 24210-346, RJ, Brazil; lean@vm.uff.br

**Keywords:** *Lachesis muta*, snake venom, Plants, *Erythroxylum ovalifolium*, *Erythroxylum subsessile*, Antivenom

## Abstract

Snake venoms are composed of a complex mixture of active proteins that induce toxic effects, such as edema, hemorrhage, and death. *Lachesis muta* has the highest lethality indices in Brazil. In most cases, antivenom fails to neutralize local effects, leading to disabilities in victims. Thus, alternative treatments are under investigation, and plant extracts are promising candidates. The objective of this work was to investigate the ability of crude extracts, fractions, or isolated products of *Erythroxylum ovalifolium* and *Erythroxylum subsessile* to neutralize some toxic effects of *L. muta* venom. All samples were mixed with *L. muta* venom, then in vivo (hemorrhage and edema) and in vitro (proteolysis, coagulation, and hemolysis) assays were performed. Overall, crude extracts or fractions of *Erythroxylum* spp. inhibited (20%–100%) toxic effects of the venom, but products achieved an inhibition of 4%–30%. However, when venom was injected into mice before the plant extracts, hemorrhage and edema were not inhibited by the samples. On the other hand, an inhibition of 5%–40% was obtained when extracts or products were given before venom injection. These results indicate that the extracts or products of *Erythroxylum* spp. could be a promising source of molecules able to treat local toxic effects of envenomation by *L. muta* venom, aiding in the development of new strategies for antivenom treatment.

## 1. Introduction

According to the World Health Organization, snake bites are considered neglected diseases and affect 5.5 million people annually, resulting in approximately 400,000 amputations and 120,000 deaths [1,2]. *Lachesis muta* (Bushmaster) is the longest venomous snake in the Americas, and the second biggest in the world. This snake is present in the equatorial forests east of the Andes, ranging from eastern Ecuador, Colombia, Peru, northern Bolivia and eastern and northern Venezuela, to Guyana, French Guyana, Surinam, and northern Brazil. In Brazil, the number of accidents with *L. muta* is lower (3%) than *Bothrops* (90%) or *Crotalus* (7%), but their mortality rates are the highest. In the Amazon rainforest, however, *L. muta* accidents account for 17% of envenomation [3,4]. *L. muta* venom consists of a mixture of toxins, including metalloproteases, serine proteases, phospholipases A_2_, l-amino acid oxidases, disintegrins, among others that cause local (pain, inflammation, tissue necrosis, edema, vomiting, diarrhea, and bleeding) and systemic effects (sweating, renal and cardiac failure, hypotension, dysphagia, hemorrhage, and shock) [3,4,5].

The official treatment for snake bites worldwide is the administration of appropriate antivenom, which has been available for more than 100 years, and up to now, is essentially performed using the same protocol. Antivenom production is carefully controlled by governments, and it is undoubtedly effective against the toxic systemic effects, but it does not neutralize the local symptoms, resulting in morbidity or disability to victims [3,6]. Moreover, antivenom therapy has other limitations, and may induce side effects (mild fever and/or anaphylaxis), requires low-temperature storage (this is critical for isolated regions without electric power), and a medical apparatus for administration of the antivenom is needed. As a result, researchers are looking for alternative treatments able to block pathophysiological effects of snake bites, and plant extracts are being thoroughly investigated with promising positive results [6,7,8]. Thus, screening plants to neutralize the deleterious effects of snake bites deserves attention. However, validation of their effectiveness in the scientific literature is poor.

The utilization of medicinal plants has been in practice over generations by indigenous and non-indigenous communities, and people who live in poor or distant areas use natural resources as a first treatment for snakebites as well as for the treatment of several pathologies and illnesses. Thus, the investigation of plant extracts as an antidote against toxic and devastating effects of snake venom is relevant [9,10,11]. Molander et al. [12] reported a protective effect of 94 different species of plants regularly used in the traditional medicine of Africa against some snake venom toxins.

Erythroxylaceae is a family of flowering trees with tropical and subtropical distribution, and four genera comprise such families: *Aneulophus* Benth, *Erythroxylum* P. Br, *Nectaropetalum* Engl., and *Pinacopodium*. *Erythroxylum* is considered the largest genus, with 230 species [13,14,15,16,17,18]. In Brazil, these species occur in two biomes: “restinga” (typical vegetation on quartzite, sandy, nutrient-poor parent materials over the Brazilian coast) and “cerrado” (savannas of central Brazil). A few studies on pharmacological and/or biological functions of *Erythroxylum* spp. have been performed. Picot et al. [19] showed that the crude extract of *E. laurifolium* had an in vivo hypoglycemia action. In addition, numerous traditional medicine applications have been reported for *Erythroxylum* species, such as an aphrodisiac, for the treatment of venereal diseases, rheumatism, arthrosis, respiratory infections, and amenorrhea [13,14,17]. Tropane alkaloids, tannins, flavonoids, and terpenes are organic compounds present in large amounts in this genus, and most of the medicinal effects have been assigned to these constituents [16,18]. However, to our knowledge, there is no data about the antivenom effect of *Erythroxylum* species.

## 2. Results and Discussion

In this work, the ability of stem extracts of *E. ovalifolium* and *E. subsessile* (extracts prepared in solvents of increasing polarities: hexane, dichloromethane, ethyl acetate, ethanol, and butanol) or products to inhibit the harmful effects (proteolysis, coagulant, hemolysis, hemorrhage, and edema) of *L. muta* venom was investigated. Overall, both plant extracts efficiently inhibited all the tested activities, but with different efficacy. However, inhibition of such activities dropped significantly for isolated products.

### 2.1. Chemical Composition of Plant Extracts

**Figure 1:**
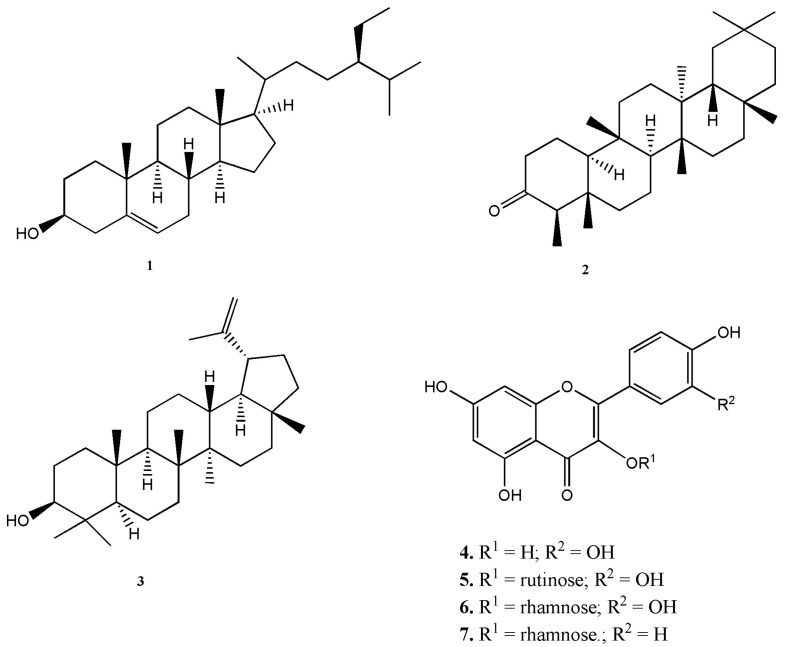
Chemical structures of metabolites of *E. subsessile* and/or *E. ovalifolium*. (**1**) β-sitosterol, (**2**) friedelin, (**3**) lupeol, (**4**) quercetin, (**5**) rutin, (**6**) quercitrin, (**7**) kaempferol-3-*O*-rhamnoside.

The plants of the genus *Erythroxylum* contain alkaloids, flavonoids, terpenes, and other metabolites with several biological and pharmacological activities [14,16,18]. The flavonoids quercetin and kaemperol are considered as chemotaxonomic markers of the *Erythroxylum* genus, as well as their 3-glycoside derivatives [20]. Phytochemical analyses of the extracts and partitions were carried out by thin-layer chromatography (TLC), nuclear magnetic resonance (NMR), gas chromatography-mass spectrometry (GC-MS), and liquid chromatography-mass spectrometry (LC-MS) in order to identify the main classes of bioactive natural products. TLC analyses of dichloromethane and hexane partitions showed the presence of terpenoids and flavonoids, while tannins were found in ethyl acetate and butanol partitions (data not shown). Alkaloids were not detected in any of the extracts by TLC analysis. Terpenoid- and flavonoid-containing partitions were selected for further fractionation based on their well known ability to inhibit the toxic activities of snake venoms [21,22]. The dichloromethane partitions of *E. ovalifolium* and *E. subsessile* were fractionated using silica gel columns and eluted in hexane: ethyl acetate gradients (10:0 to 0:10). Based on the chromatography profiles, samples were selected for a final purification with successive washes with heptane; yielding **1**. β-sitosterol (*E. subsessile* and *E. ovalifolium*), **2**. friedelin (*E. subsessile*), and **3**. lupeol (*E. ovalifolium*) (Figure 1). The ethyl acetate and butanol partitions were individually fractionated through a Sephadex LH-20 column, using ethanol as the mobile phase, and fractions were analyzed by TLC and subjected to nuclear magnetic resonance (NMR) spectroscopy, yielding: **4**. quercetin (ethyl acetate of *E. ovalifolium*), **5**. rutin (butanol of *E. ovalifolium*), **6**. quercitrin (both partitions of *E. subsessile*), and **7**. kaempferol-3-*O*-rhamnoside (both partitions of *E. subsessile*) (Figure 1). Compounds **1**–**3** were obtained as crystal, and compounds **4**–**7** were amorphous yellow solids. The isolated compounds were identified by comparison of spectroscopic data with reported values [23,24,25].

**Figure 2:**
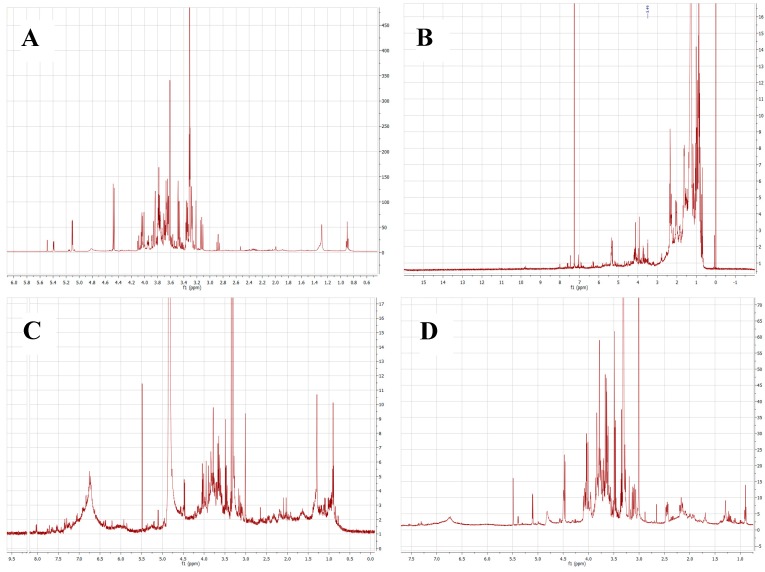
^1^H-NMR spectra of the (**A**) ethyl acetate; (**B**) hexane; (**C**) *n*-butanol; or (**D**) aqueous partitions of *E. subsessile*.

^1^H-NMR experiments were carried out in order to verify the main structural characteristics of major constituents of each sample. Since ^1^H-NMR peak area is proportional to the abundance of each atom, we focused the analysis on the major peaks of each spectrum. As seen in Figure 2, ^1^H-NMR spectra of ethyl acetate, hexane, *n*-butanol, and aqueous partitions of *E. subsessile* presented similar profiles. The spectrum of the hexane partition predominantly showed peaks of saturated hydrocarbons (δ 0.5–3.0 ppm, Figure 2B). The most deshielded peaks represent hydrogen from hydroxylated or unsaturated groups (δ 3.5–5.5 ppm). This profile was interpreted as a triterpene-rich sample. On the other hand, the major peaks obtained in the ethyl acetate spectrum are consistent with flavonoids’ aromatics rings (δ 6.0–8.0 ppm) and polyhydroxylated groups (δ 3.5–4.5 ppm, Figure 2A). These peaks were probably due to the presence of glycosylated flavonoids that are chemical markers of the genus *Erythroxylum* and are in accordance with the compounds isolated and identified in the extracts. The spectra of *n*-butanol and aqueous fractions (Figure 2C,D, respectively) presented innumerous superposition peaks between δ 6.2 and δ 8.5 ppm, possibly due to a high concentration of polyphenols of higher polarities, such as tannins. Our NMR findings are in agreement with previous works performed for different species of *Erythroxylum*. Barreiros et al. [20] demonstrated the presence of fatty acid and triterpenes as well as lupeol and β-sitosterol in the hexane partition of *E. nummularia*, and quercetin was found in the extracts of the chloroform and ethyl acetate fractions. Moreover, lupeol and β-sitosterol were detected in the hexane extract of *E. passerinum*, as well as other compounds, such as β-amyrin and erythrodiol [26]. Lupeol was identified in the methanolic leaf extract of *E. leal-costae*, in addition to other substances [27].

The ethyl acetate partitions of *E. ovalifolium* and *E. subsessile* were subjected to liquid chromatography-mass spectrometry analysis (UPLC-Orbitrap MS), and eight polyphenolic compounds were identified based on their high-resolution and accurate *m*/*z* values and MS/MS spectra provided by the Orbitrap analyzer (Table S1, Figures S1 and S2; Supplementary Materials). Compounds quercetin, quercitrin and kaempferol were found in both species, while (epi)catechin, (epi)catechin dimer (procyanidin dimer), rutin, eriodictyol-rhamnoside, and ombuin-rutinoside were found only in the ethyl acetate partition of *E. ovalifolium*. Quercitrin, ombuin-rutinoside, and eriodictyol-rhamnoside have already been described in species of the *Erythroxylum* genus [28,29,30]. Quercetin, rutin, and quercitrin were also isolated from ethyl acetate and butanol partitions.

Hexane and dichloromethane partitions from both plants were analyzed by GC-MS (Figures S4–S7 Supplementary Materials). Fridelin, friedelanol, and β-sitosterol were found in hexane and dichloromethane partitions from *E. subsessile.* β-sitosterol was found in hexane and dichloromethane partitions from *E. ovalifolium*.

Mors et al. [7] analyzed the protective effect of several plants and products against the toxic effects of *Bothrops jararaca* venom. Moreover, they investigated the mechanism of the inhibitory action of the metabolites [7]. They speculated that these metabolites may interact with active sites of enzymes through their net charge or polarity, and as a consequence, cause their inactivation [7,31]. In addition, the inhibitory action of metabolites may occur by chelating metal ions that are necessary for some enzymes in venoms to express their activities, such as PLA_2_ (Ca^2+^) and metalloproteases (Zn^2+^) [31]. These two groups of enzymes are responsible for a wide spectrum of toxic effects that follow snake bites, including hemorrhage, coagulation, necrosis, edema, and effects on platelet aggregation and hemolysis [32].

### 2.2. Antiproteolytic and Antihemolytic Effects of Plant Extracts or Products

**Figure 3:**
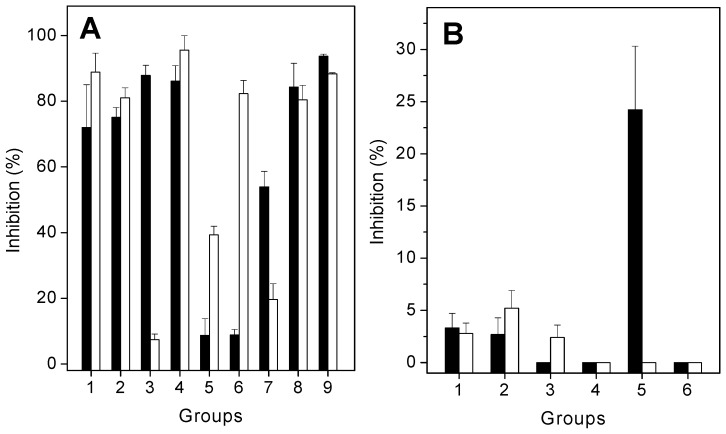
*L. muta* venom (10 µg/mL) was incubated with either: (**A**) 90 µg/mL extracts of *E. ovalifolium* in ethanol (**1**), ethyl acetate (**2**), dichloromethane (**3**), butanol (**4**), or water (**5**), or with *E. subsessile* in ethanol (**6**), hexane (**7**), ethyl acetate (**8**), dichloromethane (**9**), butanol (**10**), or water (**11**); or (**B**) with 90 µg/mL of β-sitosterol (**1**), lupeol (**2**), friedelin (**3**), quercitrin (**4**), quercetin (**5**), or rutin (**6**) for 30 min at 25 °C. Afterwards, proteolytic (black columns) or hemolytic (white columns) activities were assessed, as described in the Materials and Methods section. Data are expressed as means ± S.E. of three individual experiments (*n* = 3).

*L. muta* venom induced proteolysis or hemolysis in a concentration-dependent manner. A dose–response curve was constructed to find the effective concentration (EC) and minimum indirect hemolytic concentration (MIHD). Subsequently, these venom concentrations (10 μg/mL) were incubated with extracts, and proteolytic or hemolytic activities were investigated. As seen in Figure 3, the extracts of *E. ovalifolium* and *E. subsessile* inhibited both proteolysis (Figure 3A, black columns) and hemolysis (Figure 3A, white columns) induced by *L. muta* venom, but with different potencies. The extract of *E. ovalifolium* prepared in dichloromethane, and the ethanol, butanol, and aqueous extracts of *E. subsessile* inhibited more than 80% of proteolytic activity (Figure 3A). In addition, the ethanol, ethyl acetate, and aqueous extracts of *E. ovalifolium*, or the dichloromethane extract of *E. subsessile* inhibited proteolysis by 40% to 75%. In contrast, the hexane and ethyl acetate extracts of *E. subsessile* inhibited proteolysis by less than 10%. The hemolysis of *L. muta* venom was inhibited by more than 80% by the ethanol or ethyl acetate extracts of *E. ovalifolium*, and by the ethanol, ethyl acetate, butanol, and aqueous extracts of *E. subsessile*. In contrast, the aqueous extract of *E. ovalifolium* did not inhibit the hemolytic activity of *L. muta* venom (Figure 3A). Moreover, isolated products were also tested on proteolytic (Figure 3B, black columns) or hemolytic (Figure 3B, white columns) activities. β-sitosterol and lupeol inhibited both activities by around 5%, while quercitrin and rutin did not inhibit them. In contrast, quercetin inhibited proteolytic activity (25%), but it failed to inhibit hemolysis (Figure 3B). Neither saline nor solvents interfered with the hemolytic or proteolytic activities.

### 2.3. Anticoagulation Effect of Plants or Products

**Figure 4:**
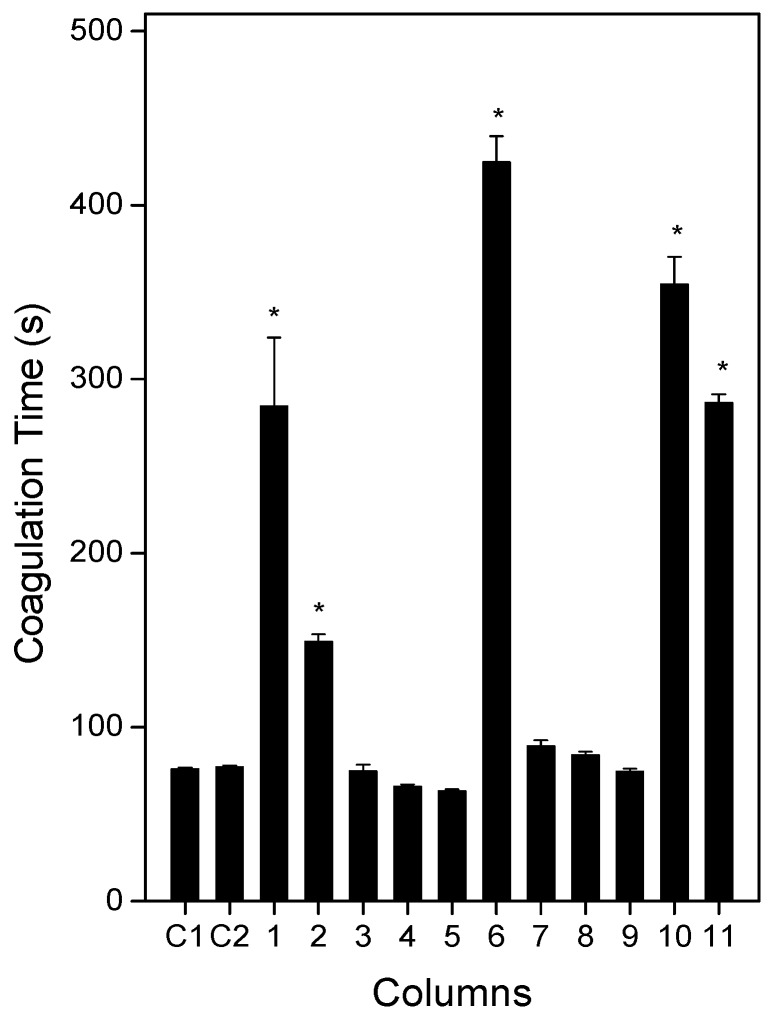
*L. muta* venom (25 µg/mL) was incubated for 30 min at 25 °C with saline (C1), DMSO (C2), or with 250 µg/mL extracts of *E. ovalifolium* in ethanol (**1**), ethyl acetate (**2**), dichloromethane (**3**), butanol (**4**), or water (**5**), and with *E. subsessile* in ethanol (**6**), hexane (**7**), ethyl acetate (**8**), dichloromethane (**9**), butanol (**10**), or water (**11**). Afterwards, mixtures were added to plasma and coagulation was performed, as described in the Materials and Methods section. Data are expressed as means ± S.E. of three individual experiments (*n* = 3). * *p* < 0.05 when compared with C1 or C2.

Disturbances in blood coagulation are a common feature in accidents caused by *L. muta* venom, and bleeding may continue for a few days, even after antivenom administration. *L. muta* venom (25 µg/mL) mixed with saline (C1) or DMSO (C2) clotted plasma in 77 s (Figure 4). Then, a similar venom concentration was incubated with the extracts of *E. ovalifolium* or *E. subsessile* (250 µg/mL) for 30 min, and the mixture was added to plasma and coagulation was monitored. As seen in Figure 4, the extracts of *E. ovalifolium* in dichloromethane, butanol, or water, as well as the extracts of *E. subsessile* in hexane, ethyl acetate, or dichloromethane did not inhibit the coagulation activity of *L. muta* venom. In contrast, the extracts of *E. ovalifolium* in ethanol or ethyl acetate and the extracts of *E. subsessile* in ethanol, butanol, or water significantly delayed plasma coagulation induced by *L. muta* venom (Figure 4). Neither of the isolated products (250 µg/mL) inhibited coagulation of *L. muta* venom. Additionally, none of the extracts or solvents alone induced coagulation of plasma.

### 2.4. Antihemorrhagic and Antiedematogenic Effects of Plants or Products

**Figure 5:**
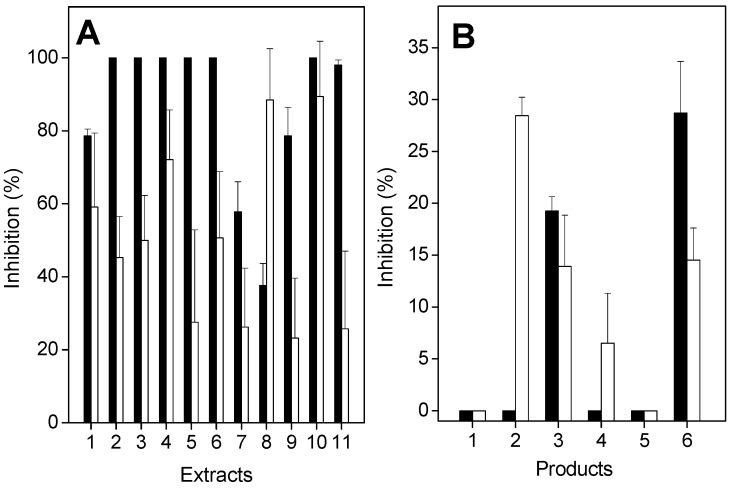
(**A**) *L. muta* venom (9 µg/mouse) was incubated for 30 min at 25 °C with 90 µg/mL extracts of *E. ovalifolium* in ethanol (**1**), ethyl acetate (**2**), dichloromethane (**3**), butanol (**4**), or water (**5**), or with *E. subsessile* in ethanol (**6**), hexane (**7**), ethyl acetate (**8**), dichloromethane (**9**), butanol (**10**), or water (**11**). Then, the mixture was injected into mice and hemorrhagic (black columns) or edematogenic (white columns) activities were assessed. (**B**) The products (90 µg/mL) β-sitosterol (**1**), lupeol (**2**), friedelin (**3**), quercitrin (**4**), quercetin (**5**), or rutin (**6**) were incubated with *L. muta* venom (black columns) or products were injected i.p. and then *L. muta* venom was injected. Then, hemorrhage was determined as described in the Materials and Methods section. Data are expressed as means ± S.E. of three individual experiments (*n* = 3).

When incubated with *L. muta* venom (9 µg/mouse), all extracts of *E. ovalifolium* or *E. subsessile* (90 µg/mL) inhibited hemorrhagic or edematogenic activity (Figure 5). The extracts of *E. ovalifolium* in ethyl acetate, dichloromethane, butanol, or water, and the extracts of *E. subsessile* in ethanol or butanol fully protected mice from the hemorrhagic effects of *L. muta* venom (Figure 5, black columns). Moreover, plant extracts also inhibited edematogenic activity of *L. muta* venom, with potencies varying from 20% to 90% (Figure 5, white columns). The extracts of *E. subsessile* prepared in ethyl acetate or butanol achieved the highest inhibitory percentage (90%) of edema, and the extracts in hexane, dichloromethane, or water had the lowest percentage (around 20%) (Figure 5A). The products β-sitosterol (**1**), lupeol (**2**), quercitrin (**4**), or quercetin (**5**) did not protect mice from hemorrhage, but frieldin (**3**) or rutin (**6**) inhibited by 20 and 28%, respectively, the hemorrhagic activity of venom (Figure 5B, black columns).

Instead of mixing *L. muta* venom with extracts or products, two other protocols were performed to mimic a real situation of envenomation. Namely: (1) treatment, in which *L. muta* venom (2 µg/mouse) was injected intradermally (i.d.) into mice, and 15 min later, the extracts of *E. ovalifolium* or *E. subsessile* (90 µg/mL) were injected at the same site of venom injection; and (2) prevention, the extracts of plants were given intraperitoneally (i.p.) or orally to animals, and 60 min later, *L. muta* venom (2 µg/mouse) was injected i.d. After both protocols (treatment or prevention), hemorrhagic and edematogenic activities were evaluated. With the treatment protocol, none of the plants inhibited hemorrhage of *L. muta* venom (data not shown). However, with the prevention protocol, both extracts inhibited hemorrhagic activity (Table 1). According to Table 1, higher inhibitory percentages of *L. muta* venom-induced hemorrhage were achieved when plants were injected i.p. (5%–40%), than by the oral route (3%–7%). In fact, most plant extracts did not inhibit hemorrhage if given orally. The ethanol extract of *E. subsessile* had the highest inhibitory percentage (40%) of hemorrhage. In contrast, when given orally, plants more efficiently protected against *L. muta* venom-induced edematogenic activity. As also seen in Table 1, the ethyl acetate or aqueous extracts of *E. subsessile* achieved the highest inhibitory percentages of edema (around 30%) if given orally to mice. However, they failed to inhibit such activity if given i.p. Moreover, the extract of *E. ovalifolium* in dichloromethane did not inhibit edema, regardless of the route of administration. With the prevention protocol, if products were injected i.p., β-sitosterol or quercetin did not inhibit hemorrhage; but an inhibition of 28% was achieved for lupeol, frieldin (14%), quercitrin (7%), or rutin (14%) (Figure 5B, white columns). On the other hand, products did not inhibit edema caused by *L. muta* venom using the prevention protocol, as some extracts did.

Flavonoids are a natural group of bioactive molecules capable of inhibiting a wide range of enzymes, such as lipoxygenase, aldose reductase, human recombinant aldose reductase, advanced glycation end products, protein tyrosine phosphatase 1B, acetylcholinesterase, butyrylcholinesterase, β-site amyloid precursor cleaving enzyme 1, inducible nitric oxide synthase, cyclooxygenase-2, and phospholipases A_2_ [33,34]. The inhibition of PLA_2_ may be the main mechanism of the antivenom activity of flavonoids. The flavonols rutin, quercetin, and quercitrin of *E. subsessile* and *E. ovalifolium* may be partially responsible for the protective effect of such plants. Moreover, flavonols isolated from *Hypericum brasiliense* or *Brownea rosademonte* have been associated with inhibition of the activity of acetylcholinesterase [35] as well as having an antivenom effect [36].

Furthermore, the antivenom effect of friedelin, lupeol, and β-sitosterol may be due to an anti-inflammatory mechanism [37], and their pentacyclic triterpene rings are responsible for such antivenom action [7]. The present study showed that the stem extracts of *E. ovalifolium* and *E. subsessile* inhibited some toxic effects (proteolysis, hemolysis, coagulation, hemorrhage, or edema) of *L. muta* venom that undoubtedly contribute to the death and morbidities of victims. In addition, the part of plant (leaf, root, stem, flour, fruit, or flowers) involved in the inhibitory effect against the toxicity of venoms as well as the solvents used to prepare extracts should be taken into consideration in studies of screening, ethnobotany, and ethnopharmacology. A previous report reinforces our results, since different inhibitory profiles were observed for the stem and leaves of *Manilkara subsericea* prepared in ethyl acetate or hexane against proteolytic, hemolytic, coagulant, hemorrhagic, and edematogenic activities of *L. muta* venom [38]. However, it is quite difficult to postulate which part of *M. subsericea* is the most active in inhibiting such activities [38]. In general, the stems of plants inhibit activities of venoms more efficiently than the flowers or leaves. Moreover, the literature shows that extracts of the *Dipteryx alata* neutralized the venoms of *B. jararacussu* and *Crotalus durissus terrificus*, and some metabolites, such as isoflavones, triterpenes, tannins, among others, have been identified as antivenom molecules [39,40,41,42]. Aside from plants, the marine alga *Canistrocarpus cervicornis* [43] and some sponges [44] also inhibited the toxic activities of venoms from *B. jararaca* and *L. muta*.

Results from the present work showed that a single part of *E. ovalifolium* or *E. subsessile* and the products of such plants were unable to block all in vivo and in vitro toxic effects of *L. muta* venom. Thus, a cocktail of different parts of plants or products should be used in order to increase the inhibitory action against the toxic effects of the venom.

## 3. Materials and Methods

### 3.1. Venom, Animals, and Reagents

*L. muta* venom was kindly supplied from Ezequiel Dias Foundation (FUNED), Belo Horizonte, Minas Gerais, Brazil. *L. muta* venom was diluted in saline (1 mg/mL) and stored at −20 °C until use. Male Swiss mice (18–20 g) were obtained from the Center of Laboratory Animals (NAL) of the Federal Fluminense University (UFF). All solvents and reagents were of the purest grade available.

### 3.2. Ethics Statement

Experiments were approved by the UFF Institutional Committee for Ethics in Animal Experimentation (protocol No. 212), in accordance with the guidelines of the Brazilian Committee for Animal Experimentation (COBEA). Plasma was obtained from human donors following the Guidelines of the Ethics Committee of the UFF Hospital, under number CEP 643.926.

### 3.3. Plant Material

The plants *E. ovalifolium* (S22 14.722′, WO41 34.916′) and *E. subsessile* (S22 16.111′, WO41 38.990′) were collected in January 2009 from the Restinga of Jurubatiba National Park, state of Rio de Janeiro, Brazil. The identification of the plants was carried out by Marcelo Guerra Santos (FFP/UERJ), and voucher samples were deposited in the Herbarium of the Rio de Janeiro State University, São Gonçalo, under the identification labels RFFP 2147 and RFFP 2126, respectively. 

### 3.4. Preparation of Plant Extracts and Partitions

Stems of *E ovalifolium* (4.2 kg) and *E. subsessile* (2.7 kg) were dried at 35 °C for 24 h with air circulation and pulverized in a grinding mill (Thomas Scientific, Swedesboro, NJ, USA). The powdered stems of *E. ovalifolium* were macerated with 96% ethanol (10% *w*/*v*) for 10 days at room temperature and filtered. The residue was macerated again to ensure the exhaustion of the drug. Subsequently, the liquid phases were pooled and filtered, the solvent fully evaporated under reduced pressure at 35 °C, and suspended in 1 L of 90% ethanol before partitioning with hexane (3 × 200 mL). The ethanol-soluble fraction was concentrated under reduced pressure, suspended in distilled water, and partitioned successively with dichloromethane (3 × 200 mL), ethyl acetate (3 × 200 mL), and butanol (3 × 200 mL). Solvents from each liquid organic phase were totally evaporated under reduced pressure at 35 °C, yielding solid hexane (32.4 g), dichloromethane (41.0 g), ethyl acetate (37.7 g), and butanol (8.6 g) partitions. The procedure was repeated for *E. subsessile*, yielding hexane (11.0 g), dichloromethane (15.5 g), ethyl acetate (16.2 g), and butanol (11.3 g) partitions. All extracts, partitions, or isolated products were dissolved in 100% dimethylsulfoxide (DMSO).

### 3.5. Phytochemical Analysis

Phytochemical investigations were performed on partitions of both plants in several steps. Firstly, samples were analyzed by TLC for detection of the main classes of natural products (i.e., flavonoids, terpenoids, tannins, and alkaloids). Secondly, partitions were fractionated by Sephadex LH-20 (GE Healthcare, Piscataway, NJ, USA) or silica gel column chromatography to separate the main constituents. One- and two-dimensional NMR spectra were recorded on a Varian VNMRS 400 MHz (Varian Inc., Santa Clara, CA, USA), using tetramethylsilane as external shift reference. The structures of isolated compounds were elucidated on the basis of ^1^H- and ^13^C-NMR spectroscopy. The partitions were also analyzed by LC-MS/MS and GC-MS. For the LC-MS/MS analysis, an UPLC-Q Exactive Plus Orbitrap mass spectrometry system (Thermo Fisher Scientific, Bremen, Germany) was employed, equipped with an electrospray ionization source operating in negative ion mode at a voltage of 2.9 kV. The column used was a Thermo Syncronis C18 (50 mm × 2.1 mm × 1.7 µm) maintained at 40 °C. The mobile phase consisted of (A) 5 mM ammonium formate and (B) methanol in gradient elution mode (0−2 min, 20% B; 2−4 min, 60% B; 4−10 min, 95% B; 10–12 min, 95% B, and 12.1−15 min, 20% B) at a flow rate of 400 μL min^−1^. GC-MS analyses were carried out using a GCMS-QP5000 (SHIMADZU) gas chromatograph equipped with a mass spectrometer using electron ionization. Conditions were as follows: injector temperature, 270 °C; detector temperature, 270 °C; carrier gas (Helium), flow rate 1 mL/min, and split injection with split ratio 1:40. Oven temperature was initially at 60 °C and then raised to 290 °C at a rate of 3 °C/min. One microliter of each sample, dissolved in CH_2_Cl_2_ (1:100 mg/μL), was injected at DB-5 column (i.d. = 0.25 mm, length 30 m, film thickness = 0.25 μm). The mass spectrometry (MS) conditions were: voltage, 70 eV; and scan rate, 1 scan/s. The identification of compounds was performed by comparing their MS fragmentation pattern with those described on NIST mass spectra library.

### 3.6. Proteolytic Activity

Proteolytic activity of *L. muta* venom was determined according to Garcia et al. [45], using azocasein as a substrate (0.2%, *w*/*v*, in 20 mM Tris-HCl, 8 mM CaCl_2_, pH 8.8). *L. muta* venom induced proteolysis in a concentration-dependent manner, and the amount of *L. muta* venom (µg/mL) able to achieve 70%–80% of maximum rate (a variation of 0.2 at absorbance 420 nm) was denoted the effective concentration (EC), and was considered as 100% of proteolytic activity. One EC of *L. muta* venom (10 µg/mL) was incubated with the plant extracts or products (90 µg/mL) for 30 min at 25 °C. After this, the mixture was incubated with 0.4 mL azocasein at 37 °C for 90 min in a total volume of 1.2 mL. The enzymatic reaction was stopped by adding trichloracetic acid (5%, *v*/*v*, final concentration), and tubes were centrifuged at 15,000× *g* for 3 min. The supernatant was then removed, mixed with 2 N NaOH, and the tubes were read at A 420 nm. As a positive control, *L. muta* venom was incubated with solvents (saline or DMSO) plus azocasein, while for negative control, extracts, products, or solvents were incubated with azocasein in the absence of *L. muta* venom.

### 3.7. Coagulating Activity

A pool of citrated normal human plasma was obtained from a local blood bank of Antônio Pedro Hospital of UFF and diluted with an equal volume in saline. *L. muta* venom (50 µL) at different concentrations (2–70 µg/mL) was added to plasma, and coagulation was monitored on an Amelung digital coagulometer, model KC4A (Labcon, Germany). The amount of venom (µg/mL) able to clot plasma in around 60 s was called the minimum coagulation dose (MCD). Then, one MCD of venom (25 µg/mL) was incubated with solvents (saline or DMSO), extracts, or products of *E ovalifolium* or *E. subsessile* (250 µg/mL) for 30 min at 25 °C, and the mixture (50 µL) was added to plasma (200 µL), and coagulation was monitored. Negative control experiments were performed in parallel by mixing plant extracts, products, or solvents with plasma in the absence of venom.

### 3.8. Hemolytic Activity

*L. muta* venom-induced hemolysis was determined through the indirect hemolytic test using human erythrocytes and hen’s egg yolk emulsion as a substrate [46]. After performing a dose–response curve (2–30 μg/mL), the amount of *L. muta* venom (μg/mL) able to produce 100% hemolysis was called the minimum indirect hemolytic dose (MIHD). The inhibitory effect of the *Erythroxylum* extracts or products was performed by incubating one MIDH of venom (10 µg/mL) with plant extracts or products (90 µg/mL) for 30 min at 25 °C, and then a hemolytic assay was performed. Control experiments were performed by incubating *L. muta* venom with solvents in the absence of plant extracts/products or by adding plant extracts, products, or solvents to the reaction medium in the absence of venom.

### 3.9. Hemorrhagic Activity

Hemorrhagic lesions produced by *L. muta* venom were quantified using a procedure described by Kondo et al. [47], with modifications. Briefly, *L. muta* venom (100 µL) was injected intradermally (i.d.) into the abdominal skin of mice. Two hours later, animals were euthanized by decapitation, the abdominal skin removed, stretched, and inspected for visual changes in the internal aspect in order to localize hemorrhagic spots. One minimum hemorrhagic dose (MHD) was defined as the amount of venom (µg/mouse) able to produce a hemorrhagic halo of 10 mm. To evaluate the inhibitory effect of plant extracts or products, different protocols were employed, as follows: (1) pre-incubation, in which the plants or products were incubated with two MHD (9 µg/mouse) of venom at 25 °C for 30 min, the mixture (100 µL) was then injected into animals, and the hemorrhagic activity was evaluated; (2) treatment, *L. muta* venom was injected i.d., and 15 min later, the plant extracts or products were injected i.d. at the same site of venom injection; and (3) prevention, plant extracts or products were administered intraperitoneally (i.p.) or orally by gavage into mice, and 60 min later, *L. muta* venom was injected i.d. into animals. For all protocols, hemorrhage was expressed as the mean diameter (in millimeters) of the hemorrhage halo induced by the venom in the absence and presence of the plant extracts or products. Negative control experiments were performed by injecting plant extracts or products i.d. or with solvents in the absence of venom.

### 3.10. Edematogenic Activity

Edematogenic activity of *L. muta* venom was determined according to Yamakawa et al. [48], with modifications. *L. muta* venom (9 µg/mouse) was injected subcutaneously (s.c.) into the right paw of mice, while the left one received solvents (DMSO or saline), plant extracts, or products (90 µg/mouse). One hour after injection, edema was evaluated as the percentage increase in weight of the right paw compared to the left one. Anti-edematogenic activity was performed by incubating 90 µg/mL of plant extracts or products with *L. muta* venom for 30 min at 25 °C, and then the mixture was injected s.c. into mice. The prevention protocol was performed, but only with some extracts or products. *E. ovalifolium* in ethanol, dichloromethane, or ethyl acetate, and *E. subsessile* in ethanol, ethyl acetate, butanol, or water were administered i.p. or orally by gavage and 60 min later, *L. muta* venom was injected s.c. into paw and edema was evaluated. Control experiments were performed by injecting solvents, plant extracts, or products in the absence of venom. The volume of injection of samples into the paw of mice was 50 µL.

### 3.11. Statistical Analysis

Results are expressed as means ± standard error (S.E.) obtained with the indicated number of animals or experiments performed. The statistical significance of differences among experimental groups was evaluated using ANOVA. Significance was taken as *p* < 0.05.

## 4. Conclusions

We showed for the first time the ability of extracts or products of *E. ovalifolium* and *E. subsessile* to inhibit the main and critical toxic activities of *L. muta* venom, indicating the potential use of these plants to treat envenomation by snake bites as well as to study the mechanism of inhibitory action of products. This work also highlights the fact that Brazilian flora is a rich source of molecules with antivenom properties, deserving a deeper prospecting research of such plants.

## Figures and Tables

**Table 1:** Effect of *E. ovalifolium* or *E. subsessile* on hemorrhage and edema induced by *L. muta* venom.

Sample	% Inhibition			
	Hemorrhage		Edema	
	i.p.	oral	i.p.	oral
**1**	8 ± 9	0	0	22 ± 7
**2**	5 ± 7	0	18 ± 9	19 ± 9
**3**	10 ± 8	3 ± 15	0	0
**4**	40 ± 4	4 ± 12	12 ± 6	17 ± 4
**5**	7 ± 9	0	0	29 ± 8
**6**	18 ± 5	0	12 ± 4	5 ± 2
**7**	14 ± 9	7 ± 1	0	30 ± 9

The extracts (90 µg/mL) of *E. ovalifolium* in ethanol (**1**), ethyl acetate (**2**), or dichloromethane (**3**), and the extracts of *E. subsessile* in ethanol (**4**), ethyl acetate (**5**), butanol (**6**), or water (**7**) were administered intraperitoneally or orally into mice. Then, 60 min later, *L. muta* venom (9 µg/mouse) was injected into mice and hemorrhage and edema were evaluated, as described in the Materials and Methods section. Data are expressed as means ± S.E. of three individual experiments (*n* = 3).

## Supplementary Materials

Supplementary materials can be accessed at: http://www.mdpi.com/1420-3049/21/10/1350/s1.

## Abbreviations

The following abbreviations are used in this manuscript:

DMSO: Dimethyl sulfoxide
MCD: Minimum Coagulation Dose
MHD: Minimum Hemorrhagic Dose
MIHD: Minimum Indirect Hemolytic Dose
PLA_2_: Phospholipase A_2_
TLC: Thin-layer chromatography
*E. ovalifolium*: *Erythroxylum ovalifolium*
*E. subsessile*: *Erythroxylum subsessile*
*L. muta*: *Lachesis muta*
