# Supplementary material for: Protective Effect of the Plant Extracts of Erythroxylum sp. against Toxic Effects Induced by the Venom of Lachesis muta Snake
Source: Molecules. 2016 Oct 11;21(10):1350. doi: 10.3390/molecules21101350 (PMC6274453; doi:10.3390/molecules21101350)
Supplement: Supplementary file 1 [file molecules-21-01350-s001.pdf]

Supplementary Materials: Protective Effect of the Plant Extracts of *Erythroxylum* sp. against Toxic Effects Induced by the Venom of *Lachesis muta* Snake

Eduardo Coriolano de Oliveira, Rodrigo Alves Soares Cruz, Nayanna de Mello Amorim, Marcelo Guerra Santos, Luiz Carlos Simas Pereira Junior, Eládio Oswaldo Flores Sanchez, Caio Pinho Fernandes, Rafael Garrett, Leandro Machado Rocha and André Lopes Fuly

Table S1. Compounds identified by UPLC-Orbitrap MS in the ethyl acetate partition of *Erythroxylum ovalifolium* and *E. subsessile.*

Compounds	Rt (min)	*m/z* $[M-H]^-$	MS/MS	Molecular Formula	Error (ppm)	Ref.
			**Erythroxylum ovalifolium**			
Procyanidin dimer	0.8	577.13660	407, 289, 245, 161, 125	$C_{30}H_{26}O_{12}$	1.8	[1]
(Epi)catechin	1.4	289.07188	203, 179, 125, 109	$C_{15}H_{14}O_6$	0.8	[1]
Rutin	5.0	609.14611	301, 179, 151	$C_{27}H_{30}O_{16}$	1.2	[2]
Eriodictyol-rhamnoside	5.3	433.11449	287, 179, 151	$C_{21}H_{22}O_{10}$	1.1	[3]
Quercitrin *	5.5	447.09328	301, 179, 151	$C_{21}H_{20}O_{11}$	2.1	[4]
Quercetin *	6.2	301.03532	273, 179, 151, 121	$C_{15}H_{10}O_7$	0.4	[1]
Kaempferol *	6.4	285.04046	175, 161, 151	$c_{15}H_{10}O_6$	0.8	[2]
Ombuin-rutinoside	6.7	637.17761	329, 314, 299	$C_{29}H_{34}O_{16}$	0.5	[4]

* Compounds also found in *Erythroxylum subsessile.*

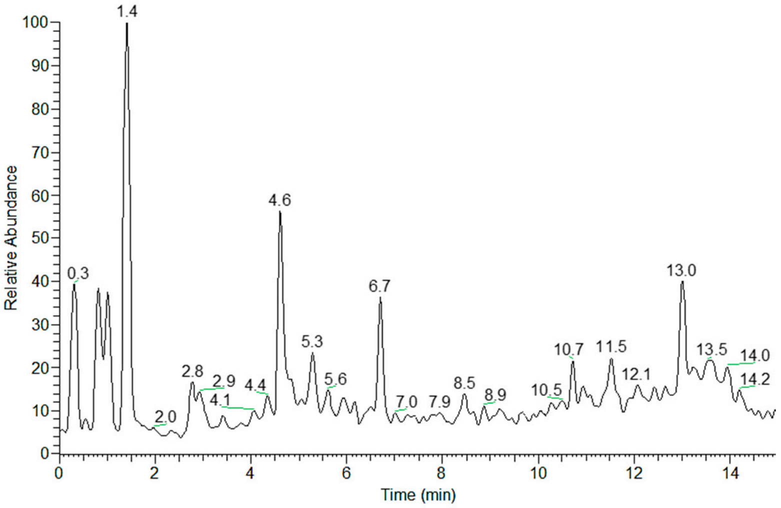

Figure S1. Total ion chromatogram of the ethyl acetate partition of *Erythroxylum ovalifolium* by UPLC-ESI-Orbitrap MS in negative ion mode.

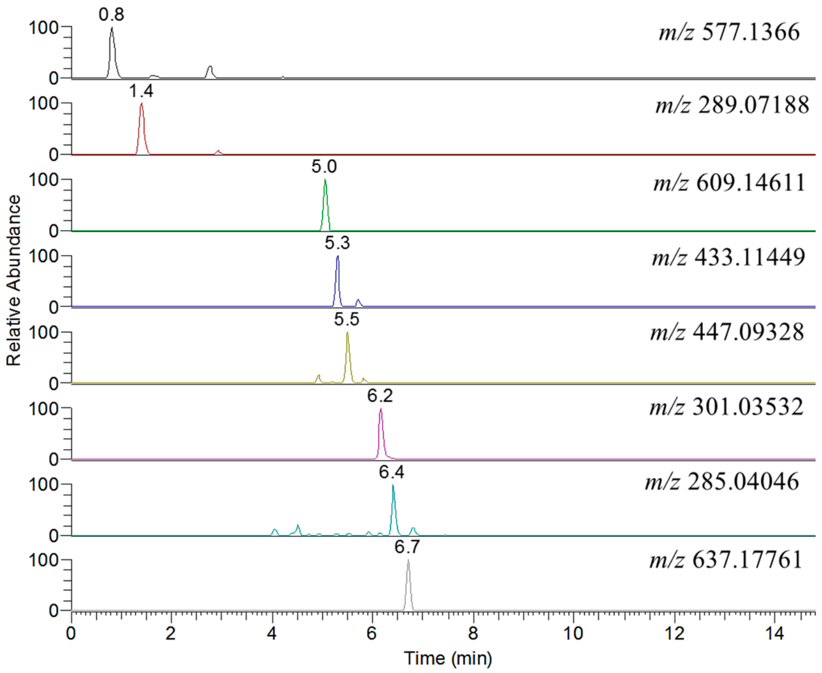

Figure S2. Extracted-ion chromatograms of the ethyl acetate partition of *Erythroxylum ovalifolium* by UPLC-ESI-Orbitrap MS in negative ion mode.

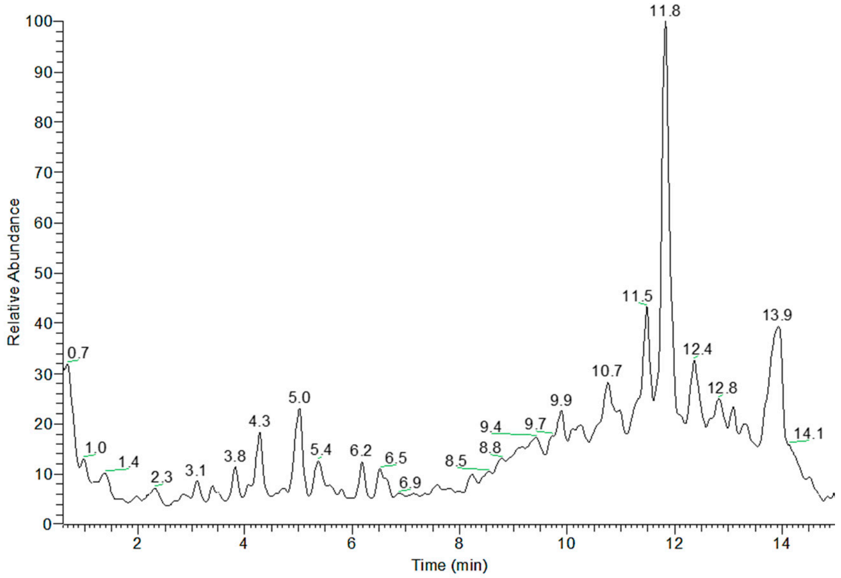

Figure S3. Total ion chromatogram of the ethyl acetate partition of *Erythroxylum subsessile* by UPLC-ESI-Orbitrap MS in negative ion mode.

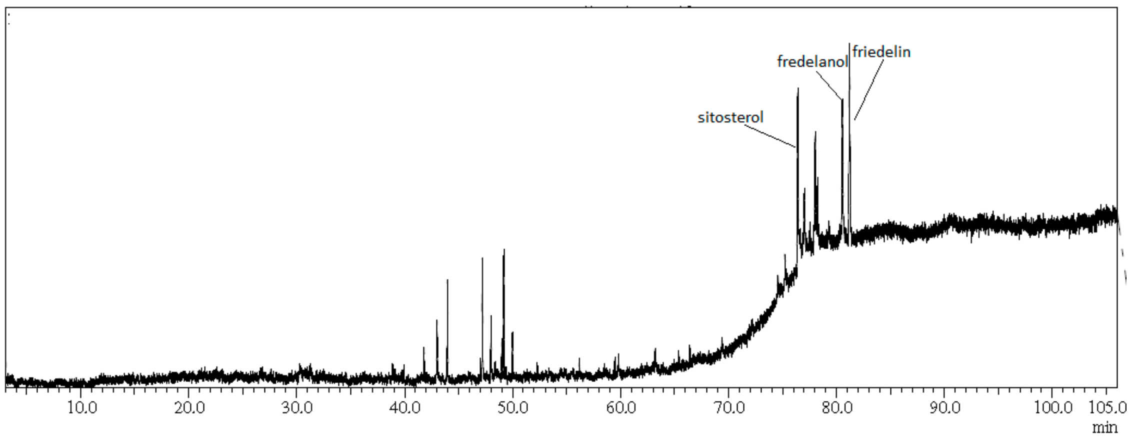

Figure S4. Total ion chromatogram of the hexane partition of *Erythroxylum subsessile* by GC-MS.

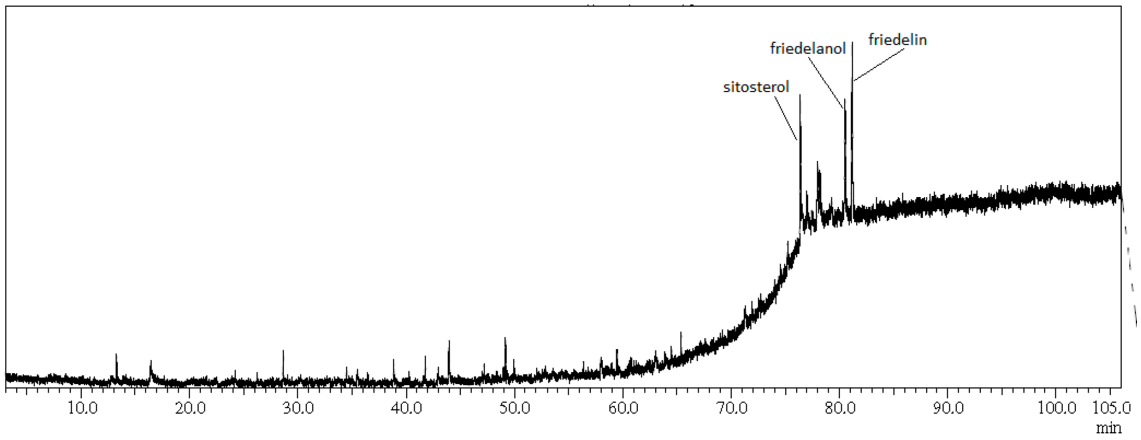

Figure S5. Total ion chromatogram of the dichloromethane partition of *Erythroxylum subsessile* by GC-MS.

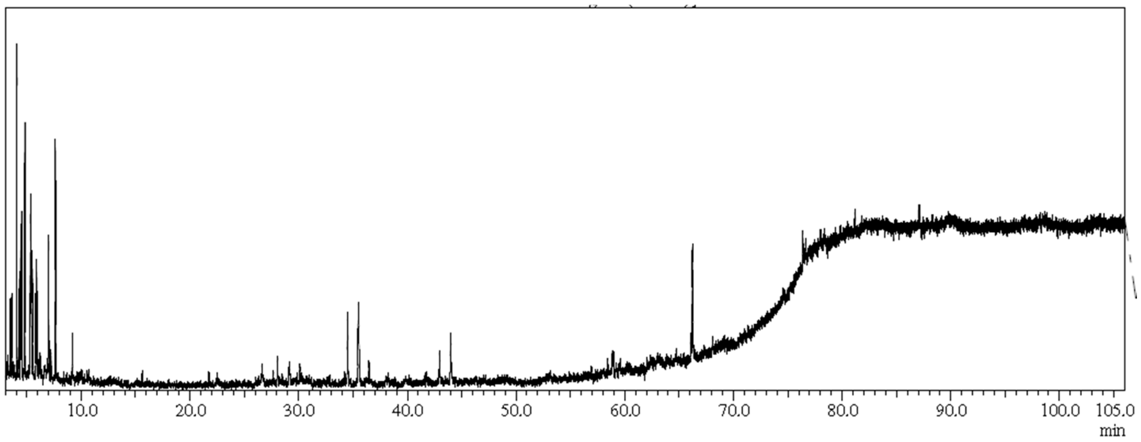

Figure S6. Total ion chromatogram of the hexane partition of *Erythroxylum ovalifolium* by GC-MS.

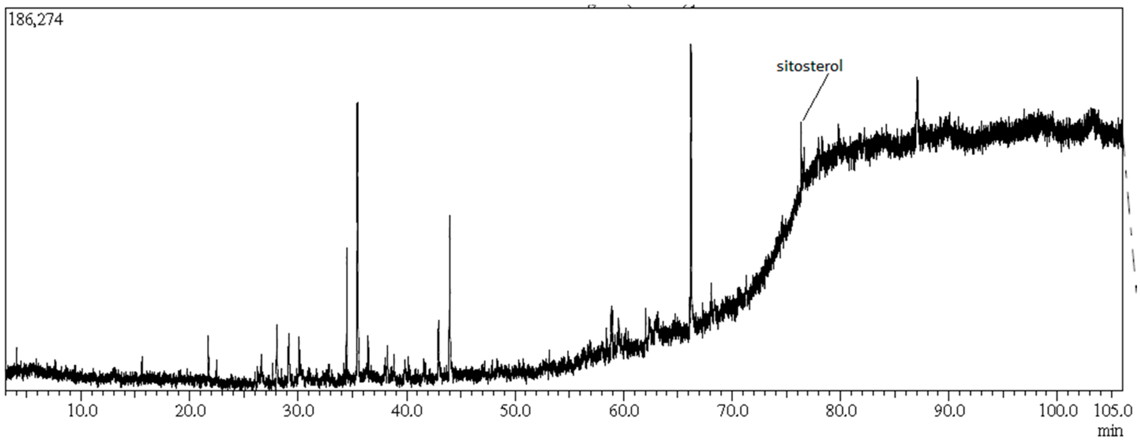

Figure S7. Total ion chromatogram of the dichloromethane partition of *Erythroxylum ovalifolium* by GC-MS.

References

1. Souza, L.M.; Cipriani, T.C.; Iacomini, M.; Gorin, P.A.J.; Sassaki, G.L. HPLC/ESI-MS and NMR analysis of flavonoids and tannins in bioactive extract from leaves of *Maytenus ilicifolia*. *J. Pharm. Biomed. Anal.* **2008**, *47*, 59–67.
2. Brito, A.; Ramirez, J.E.; Areche, C.; Sepúlveda, B.; Simirgiotis, M. J. HPLC-UV-MS Profiles of phenolic compounds and antioxidant activity of fruits from three citrus species consumed in Northern Chile. *Molecules* **2014**, *19*, 17400–17421.
3. Johnson, E.L.; Schmidt, W.F.; Cooper, D. Flavonoids as chemotaxonomic markers for cultivated Amazonian coca. *Plant Physiol. Biochem.* **2002**, *40*, 89–95.
4. Albuquerque, C.H.; Tavares, J.J.; de Oliveira, S.L.; Silva, T.S.; Gonçalves, G.R.; Costa, V.C.; Agra, M.F.; Pessôa, H.L.F.; da Silva, M.S. Flavonoides glicosilados de *Erythroxylum pulchrum* A. St.-Hil. (Erythroxylaceae). *Quim. Nova* **2014**, *37*, 633–666.
